# High-yield bioactive triterpenoid production by heterologous expression in *Nicotiana benthamiana* using the Tsukuba system

**DOI:** 10.3389/fpls.2022.991909

**Published:** 2022-08-18

**Authors:** Jutapat Romsuk, Shuhei Yasumoto, Ery Odette Fukushima, Kenji Miura, Toshiya Muranaka, Hikaru Seki

**Affiliations:** ^1^Department of Biotechnology, Graduate School of Engineering, Osaka University, Suita, Osaka, Japan; ^2^Industrial Biotechnology Initiative Division, Institute for Open and Transdisciplinary Research Initiatives, Osaka University, Suita, Osaka, Japan; ^3^Plant Translational Research Group, Universidad Regional Amazónica IKIAM, Tena, Ecuador; ^4^Tsukuba-Plant Innovation Research Center, University of Tsukuba, Tsukuba, Ibaraki, Japan

**Keywords:** plant-specialized metabolites, oleanolic acid, transient protein expression, CYP716A12, *Nicotiana benthamiana*, triterpenoids

## Abstract

Oleanolic acid is a pentacyclic triterpenoid found in numerous plant species and is a precursor to several bioactive triterpenoids with commercial potential. However, oleanolic acid accumulates at low levels in plants, and its chemical synthesis is challenging. Here, we established a method for producing oleanolic acid in substantial quantities *via* heterologous expression of pathway enzymes in *Nicotiana benthamiana*. The “Tsukuba system” is one of the most efficient agroinfiltration-based transient protein expression systems using the vector pBYR2HS, which contains geminiviral replication machinery and a double terminator for boosting expression. Additionally, the pBYR2HS vector contains an expression cassette for the gene-silencing suppressor p19 protein from tomato bushy stunt virus, which can also contribute to enhancing the expression of target proteins. In this study, we evaluated the applicability of this system to heterologous triterpenoid production in *N*. *benthamiana. Medicago truncatula* cytochrome P450 monooxygenase (CYP) 716A12 is the first enzyme to be functionally characterized as β-amyrin C-28 oxidase producing oleanolic acid. A mutant CYP716A12 (D122Q) with improved catalytic activity engineered in our previous study was co-expressed with other enzymes in *N*. *benthamiana* leaves. Using pBYR2HS, oleanolic acid yield was increased 13.1-fold compared with that using the conventional binary vector, indicating the advantage of the Tsukuba system. We also demonstrated the efficacy of co-expressing a mutant *Arabidopsis thaliana* HMGR1 catalytic domain, additional NADPH-cytochrome P450 reductase (CPR) transferring electrons to heterologous CYPs, and application of ascorbic acid for preventing leaf necrosis after agroinfiltration, to improve product yield. As a result, the product yields of both simple (β-amyrin) and oxidized (oleanolic acid and maslinic acid) triterpenoids were significantly improved compared with the previously reported yield in heterologous triterpenoid production in *N*. *benthamiana* leaves.

## Introduction

Oleanolic acid is a naturally occurring pentacyclic triterpenoid found in more than 200 species of plants. Numerous studies have indicated that it is a precursor of a variety of bioactive triterpenoids of commercial interest, including bardoxolone methyl, a potent activator of the Nrf2 pathway and inhibitor of the NF-κB pathway, currently in a phase 2 clinical trial for the treatment of chronic diseases (Liby and Sporn, 2012). Oleanolic acid is also a precursor of onjisaponin F, which increases nasal anti-influenza virus IgA antibody titers (Li et al., 2016; Peng et al., 2020); platycodin D, which has antitumor and antiviral properties (Jeon et al., 2019; Kim et al., 2021); and maslinic acid, which is currently being studied as a new therapeutic agent with various activities, including anti-inflammatory, hepatoprotective, analgesic, antimicrobial, antimycotic, virostatic, and immunomodulatory activities (Fukumitsu et al., 2016; Reyes-Zurita et al., 2016). However, oleanolic acid accumulates at low levels in plants, and chemical synthesis is difficult due to its complex structure (Dale et al., 2020).

Oleanolic acid is biosynthesized from 2,3-oxidosqualene *via* cyclization catalyzed by oxidosqualene cyclase (OSC), i.e., β-amyrin synthase (bAS), to produce β-amyrin, followed by three-step oxidation at C-28 of the β-amyrin backbone through erythrodiol (28-hydroxy-β-amyrin) and oleanolic aldehyde as reaction intermediates. This oxidation reaction is catalyzed predominantly by cytochrome P450 monooxygenases (CYPs) belonging to the CYP716A subfamily (Fukushima et al., 2011; Suzuki et al., 2018). The CYP716A subfamily is a member of the CYP716 family of enzymes with highly conserved functionality in triterpenoid biosynthesis; the most common modification of the triterpene backbone catalyzed by CYP716A is three-step site-specific oxidation at the C-28 position of α-amyrin, β-amyrin, and lupeol (Fukushima et al., 2011, 2013; Miettinen et al., 2017). *Medicago truncatula* CYP716A12 was the first enzyme in the CYP716A subfamily to be functionally characterized as a multifunctional triterpene oxidase capable of producing these C-28-oxidized products (Carelli et al., 2011; Fukushima et al., 2011).

Previously, we identified the key amino acid residues responsible for determining catalytic activity and substrate specificity of CYP716A12 by functional analysis using engineered *Saccharomyces cerevisiae* producing the β-amyrin backbone (Romsuk et al., 2022). When key amino acid residues in substrate recognition sites were replaced, the product profiles of CYP716A12 were changed. Three mutant CYP716A12s (D122Q, Q358P, D122Q_Q358P) showed improved product yield for oleanolic acid, whereas two others (I212P and I212P_Q358P) predominantly produced erythrodiol.

A synthetic biology technique involving pathway reconstruction in heterologous hosts, such as yeast and *Nicotiana benthamiana*, has been investigated as an alternative method of triterpenoid production (Fukushima et al., 2011; Buyel et al., 2017; Reed et al., 2017; Suzuki et al., 2018; Dale et al., 2020). Transient protein expression in *N*. *benthamiana* leaves *via* agroinfiltration is a simpler, more flexible, and cost-effective method for studying cell biology and physiology, as well as for producing high levels of recombinant proteins than stable transgenic approaches or traditional cell culture-based systems (Buyel et al., 2017; Suzaki et al., 2019). Transient expression experiments in *N*. *benthamiana* have also led to the reconstruction of various natural product pathways. *N*. *benthamiana* has recently emerged as a suitable host for the production of terpenes, including triterpenoids (Reed et al., 2017; De La Pena and Sattely, 2021). An efficient and high-level transient expression system should produce a high yield of the desired compound. The Tsukuba system is one of the most efficient agroinfiltration-based transient protein expression systems for plant cells using the high-expression binary vector pBYR2HS (Yamamoto et al., 2018). This vector, which contains geminiviral replication machinery and a double terminator, has been shown to significantly increase transient protein expression efficiency in lettuce, *N*. *benthamiana*, tomatoes, eggplant, hot pepper, melon, and orchid. The double terminator enhances the expression level compared to a single terminator by eliminating transcriptional interference, and the geminiviral replication machinery results in a high yield of foreign protein expression (Yamamoto et al., 2018; Suzaki et al., 2019). However, it has not yet been applied to reconstitute plant triterpenoid pathways.

In this study, we evaluated the applicability of the Tsukuba system for triterpenoid production in *N*. *benthamiana* through pathway reconstruction. By comparing the pBYR2HS-based expression construct to the conventional binary vector without the geminiviral replication machinery, double terminator, we demonstrated the efficacy of pBYR2HS in increasing oleanolic acid production. We also chose the host based on the performance of two *Nicotiana* species in producing oleanolic acid. The optimal incubation time and application of ascorbic acid following agroinfiltration were also determined to prevent leaf necrosis and thus increase oleanolic acid production. In addition, we demonstrated the requirement for an additional NADPH-cytochrome P450 reductase (CPR) to optimize the microenvironment of CYPs and increase oleanolic acid production. We selected five mutant CYP716A12s that showed altered product profiles based on a previous study (Romsuk et al., 2022) as well as several wild-type CYP716A enzymes, including *Vitis vinifera* CYP716A15, *Olea europaea* CYP716A48, and *Beta vulgaris* CYP716A49, to compare the potential for high-titer oleanolic acid production using the Tsukuba system. The mutant enzyme CYP716A12_D122Q had the highest potential for heterologous oleanolic acid production. We also optimized the biosynthetic pathway to increase oleanolic acid production by co-introduction of mutant *Arabidopsis thaliana* HMGR1 catalytic domain (AtHMGR1cd-S577A) to boost the mevalonate pathway. Finally, we demonstrated the adaptability of this expression system for increasing yield in the bioactive triterpenoid pathway by highlighting maslinic acid as a key example.

This study established a general strategy for rapidly producing substantial quantities of triterpenoids and generating valuable and difficult-to-obtain triterpenoids with potent biological activity.

## Materials and methods

### Chemical authentic standards

All triterpenoid standards (uvaol, β-amyrin, erythrodiol, oleanolic acid, and maslinic acid) were purchased from Extrasynthese (Genay, France).

### Plasmid construction

pRI 201-AN (TaKaRa Bio, Shiga, Japan), designed for high-level foreign gene expression in dicotyledonous plants, was used as a conventional binary vector in this study. To construct a gateway-compatible version of pRI 201-AN, a gateway cassette was inserted into pRI 201-AN digested with NdeI and SacI to generate pYS_015 (Srisawat et al., 2019). pRI201-AN was also used as the template vector for cassette amplification to construct pBYR2HS (Yamamoto et al., 2018). The structures of T-DNA regions of pBYR2HS and pRI 201-AN-based expression constructs are shown in Supplementary Figure 1. To construct a gateway-compatible version of pBYR2HS, pBYR2HS-SalI (Yamamoto et al., 2018) was digested with SalI, blunted with KOD polymerase, and ligated with GATEWAY conversion cassette frame A (Invitrogen, Carlsbad, CA, United States).

The entry clones of pENTR-CYP716A12, pENTR-selected mutant CYP716A12s, pENTR-CYP716A15, pENTR-CYP716A48, pENTR-CYP716A49, mutant *A. thaliana* HMGR1 catalytic domain (AtHMGR1cd-S577A), and *Lotus japonicus* NADPH-cytochrome P450 reductase class II (LjCPR2) were obtained in previous studies (Seki et al., 2008; Fukushima et al., 2011; Robertlee et al., 2018; Suzuki et al., 2018; Istiandari et al., 2021). The amino acid sequences of the enzymes are shown in Supplementary Method 1. The mutant CYP716A12s were generated by site-directed mutagenesis using a PrimeSTAR® Mutagenesis Basal Kit (TaKaRa Bio) in a previous study (Romsuk et al., 2022). We synthesized a complete codon-optimized *L. japonicus* β-amyrin synthase (LjOSC1) for expression in *N*. *benthamiana* (Supplementary Method 2). The coding sequences (CDSs) of the target genes were transferred to gateway-compatible versions of pBYR2HS and pYS_015 using Gateway LR Clonase II Enzyme Mix (Thermo Fisher Scientific, Waltham, MA, United States) to generate plasmids for transient expression in plant cells.

### Transient expression in *Nicotiana benthamiana*

Expression constructs for target genes were introduced into *Agrobacterium tumefaciens* strain GV3101 (pMP90) by electroporation (MicroPulser™; Bio-Rad, Hercules, CA, United States). Transformants were selected by incubation for 3 days at 28°C on LB selection medium containing 50 mg/l kanamycin, 25 mg/l gentamycin, and 100 mg/l rifampicin. Each selected transformant was cultured in 2 ml LB selection medium at 28°C overnight with shaking at 200 rpm. Aliquots of 75 μl of the overnight cultures were transferred into 5 ml of the same medium and then cultured at 28°C overnight with shaking at 200 rpm. Freshly grown *A*. *tumefaciens* cells were resuspended in infiltration buffer (10 mM MgCl_2_, 10 mM MES, pH 5.6, 100 μM acetosyringone) to adjust OD_600_ to ~1 as described previously (Yamamoto et al., 2018). Equal volumes of *A*. *tumefaciens* harboring target constructs were mixed and infiltrated into the abaxial air spaces of the leaves of 5-week-old *N*. *benthamiana* plants using a needleless 1 ml syringe.

To prevent necrosis, we applied 0.3 ml of 200 mM ascorbic acid or 200 mM ascorbic acid sodium salt to each leaf at 2-day intervals (i.e., on days 1, 3, and 5) post-infiltration. The leaves of three plants were harvested for triterpenoid analysis at 7 days post-infiltration.

### Metabolite extraction of *Nicotiana benthamiana* leaves

To quantify triterpenoid production in the *N*. *benthamiana* leaves, 20 μl internal standard (uvaol, 100 ppm in methanol) was added to the powdered freeze-dried leaf samples before extraction. The samples (10 mg) were extracted with 1 ml methanol (Wako, Osaka, Japan). Then the mixtures were vortexed and sonicated for 60 min (45% intensity; Sharp, Osaka, Japan). After centrifugation at 12,000 rpm for 5 min, the organic phase was transferred to a fresh tube using a Pasteur pipette. The extracted samples were evaporated using a centrifugal evaporator for 45 min or until they were dry. The remaining powder was resuspended in 1 ml methanol. To remove macromolecules such as sugar and chlorophyll from the extracted sample, saponification was performed by adding 1 ml methanol and 1 ml 4 M HCl. The mixtures were briefly vortexed, heated at 80°C for 1 h, and left to stand at room temperature for 10 min. The final extraction step was performed by adding 2 ml ethyl acetate and hexane (1:1 v/v). After centrifugation at 1500 rpm for 3 min, the organic phase was transferred to a fresh tube using a Pasteur pipette. The extracted samples were evaporated using a centrifugal evaporator for 60 min or until they were dry. The remaining powder was resuspended in 500 μl methanol. The obtained samples were transferred into vials using a Pasteur pipette and stored at 4°C until analysis.

### GC–MS analysis of leaf extracts and standards

To quantify triterpenoid production in *N*. *benthamiana*, 50 μl sample solution was transferred to vial inserts and then to a centrifugal evaporator for 60 min or until dry. Then the evaporated pellet was derivatized with 50 μl *N*-methyl-*N*-(trimethylsilyl) trifluoroacetamide (Sigma-Aldrich, St. Louis, MO, USA) for 30 min at 80°C before GC–MS analysis. Aliquots of 50 μl authentic standard solutions (uvaol, β-amyrin, erythrodiol, oleanolic acid, maslinic acid, 10 ppm in methanol) were applied using the same approach as described above. GC–MS analysis was performed using a 5977A MSD coupled with a 7890B GC system and HP-5MS capillary column (length 30 m, 0.25 mm internal diameter, 0.25 μm film thickness; Agilent Technologies, Santa Clara, CA, United States). The injection component and the MSD transfer line were set to 250°C. The oven temperature was programmed as follows: 80°C for 1 min, followed by an increase to 300°C at a rate of 20°C min^−1^, and held at 300°C for 18 min. The carrier gas was helium (He) and the flow rate was 1 ml min^−1^.

Mass spectra were acquired by scanning the 50–750 m/z range. The retention times and mass spectra of the peaks were compared to those of authentic standards. The relative concentrations of triterpenoids in extracted samples were determined by comparing the peak areas of the analyte and internal standard. The concentrations of triterpenoids in the *N*. *benthamiana* leaves were determined by comparison with authentic standard curves constructed using β-amyrin, erythrodiol, oleanolic acid, and maslinic acid (Supplementary Method 3; Supplementary Figure 2).

### Statistical analysis

The differences in oxidized triterpenoid concentrations between samples extracted from each combination or condition were determined by one-way analysis of variance (ANOVA), and the significance of the means was determined using Tukey’s test. The differences in oxidized triterpenoid levels between the two groups were compared using the unpaired Student’s *t*-test. Statistical analyses were performed using JASP 0.16 on macOS (JASP Team, University of Amsterdam, Amsterdam, the Netherlands). In all analyses, *p* < 0.05 was taken to indicate statistical significance.

## Results

### Establishing a method for improving oleanolic acid production

To compare the yield of oleanolic acid in transiently transfected *N*. *benthamiana* leaves harboring pBYR2HS-based expression construct to the conventional pYS_015-based expression construct, leaves were infiltrated with a mixture of *A*. *tumefaciens* harboring pBYR2HS expressing LjOSC1 (β-amyrin synthase), LjCPR2, and CYP716A12_D122Q or a combination of pYS_015 vector expressing LjOSC1, LjCPR2, CYP716A12_D122Q, and p19 by infiltration. Given that the pBYR2HS vector contains an expression cassette for p19, we included a separate p19 expression construct in the pYS_015 vector-based infiltration mixture.

**Figure 1:**
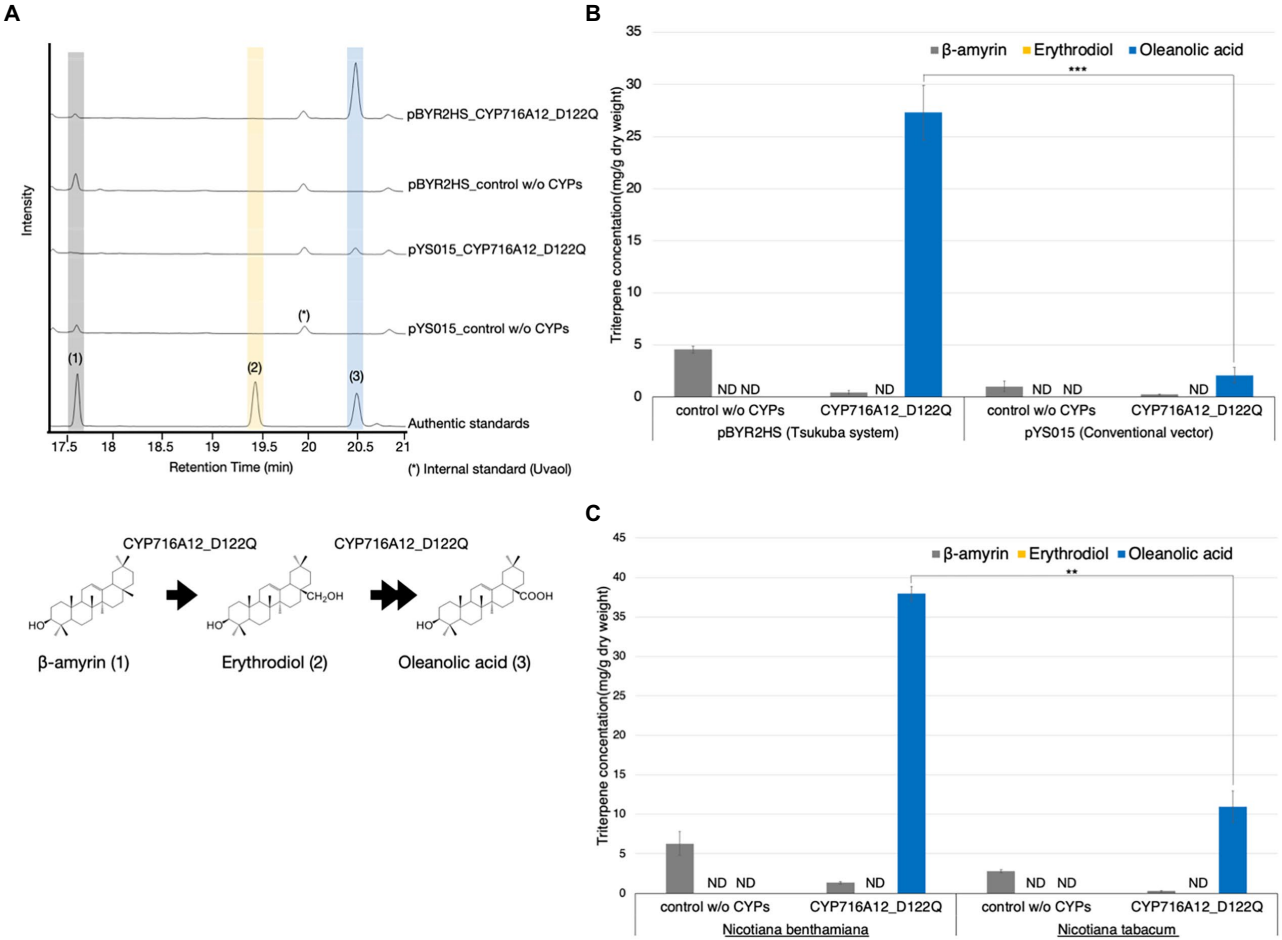
Comparison of the accumulation of oleanolic acid in leaves from different expression systems and *Nicotiana* species. **(A)** Total ion chromatograms (TICs) of extracts from *Nicotiana benthamiana* leaves infiltrated with a mixture of *Agrobacterium tumefaciens* harboring pBYR2HS expressing LjOSC1, LjCPR2, and CYP716A12_D122Q or a combination of the pYS_015 vector expressing LjOSC, LjCPR2, CYP716A12_D122Q, and p19. As a background control, a combination of pBYR2HS expressing LjOSC, LjCPR2, and empty vector or pYS015 binary vector expressing LjOSC1, LjCPR2, empty vector, and p19 was used. β-Amyrin (**1**), erythrodiol (**2**), and oleanolic acid (**3**) were used as authentic standards. Uvaol (*) was used as an internal standard. **(B)** Comparison of triterpene concentrations in *N*. *benthamiana* leaves infiltrated with different expression vectors. **(C)** Comparison of the triterpene concentrations in infiltrated leaves of *N*. *benthamiana* and *N*. *tabacum*. Data are representative of at least three biological replicates (*n* = 3) and are expressed as the mean ± SEM. Differences in oleanolic acid production were examined using Student’s *t*-test for each sample. ^**^p < 0.01; ^***^p < 0.001; ND, not detected.

As a background control, a combination of pBYR2HS expressing LjOSC1, LjCPR2, and empty vector or pYS_015 vector expressing LjOSC1, LjCPR2, empty vector, and p19 was used. We observed a single major product peak in the gas chromatogram of the extracts of *N*. *benthamiana* leaves transiently expressing CYP716A12_D122Q but not in the background control (Figure 1A). This peak was identified as oleanolic acid (**3**) by comparison with the authentic standard (Figure 1A). We quantified the triterpenoid contents of *N*. *benthamiana* leaf extracts to compare the accumulation of oleanolic acid (Figure 1B). When pYS_015 (conventional binary vector) was used, the accumulation of oleanolic acid was determined as 2.1 ± 0.7 mg/g dry weight (dw). Interestingly, the accumulation of oleanolic acid was increased 13.1-fold (27.3 ± 2.6 mg/g dw) when the pBYR2HS (Tsukuba system) vector was used (Figure 1B).

The transformation of *Nicotiana* species, specifically *N. tabacum* and *N*. *benthamiana*, with *Agrobacterium* using leaf disks as the target explant is an extremely useful method for rapid evaluation of transgenes in higher plants (Clemente, 2006). To select the better *Nicotiana* species for producing oleanolic acid using agroinfiltration-based transient protein expression, equal volumes of *A*. *tumefaciens* harboring expression vectors expressing LjOSC1, LjCPR2, and CYP716A12_D122Q were infiltrated into *N*. *benthamiana* and *N*. *tabacum*.

GC–MS was performed to determine the amounts of oleanolic acid in infiltrated leaves of different *Nicotiana* species (Supplementary Figure 3). *Nicotiana benthamiana* leaves yielded 37.9 ± 0.9 mg/g dw oleanolic acid, which was 3.3-fold higher than that in *N. tabacum* leaves (11.4 ± 1.7 mg/g dw; Figure 1C). Our findings indicate that both *N. benthamiana* and *N. tabacum* could be used as heterologous hosts for producing oleanolic acid using the Tsukuba system. However, in terms of product yield per leaf, our observations tend to indicate that of the two species, *N. benthamiana* would appear to provide a more promising production platform.

### Requirement of additional CPR for optimization of the CYP microenvironment to enhance oleanolic acid production

**Figure 2:**
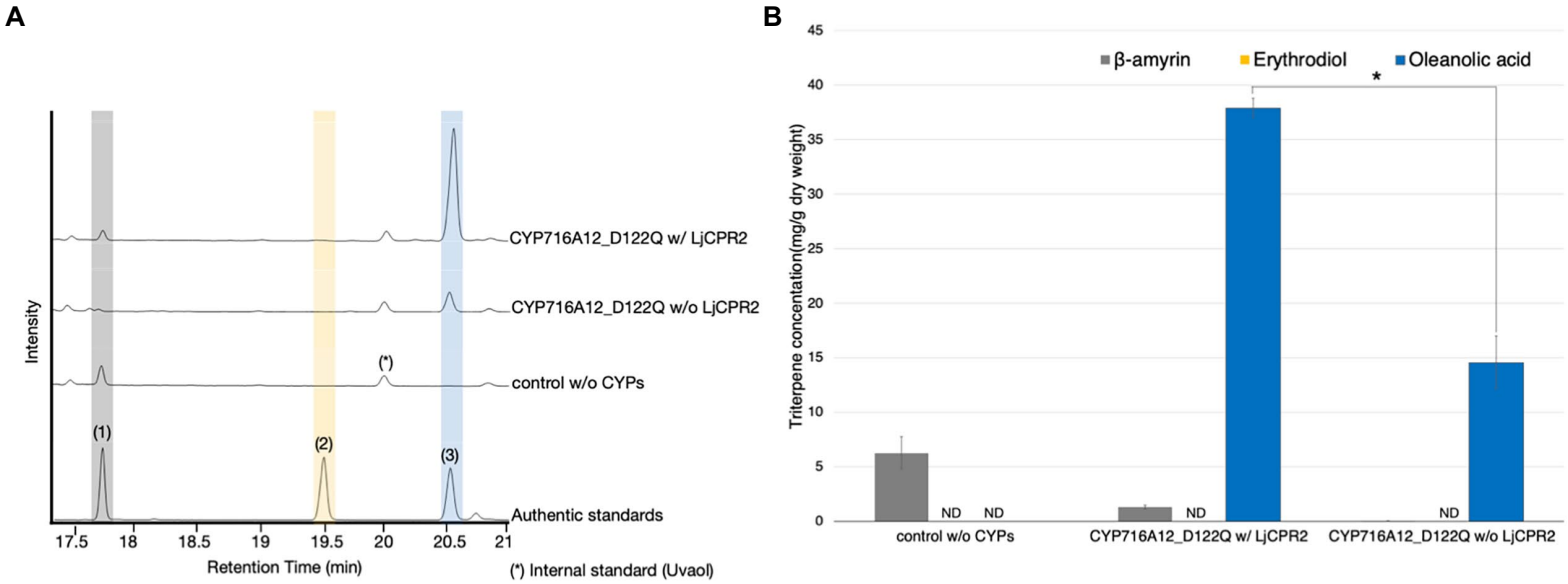
Requirement of additional CPR to optimize the CYP microenvironment for enhancement of oleanolic acid production in *Nicotiana benthamiana.* **(A)** TICs of extracts from *N*. *benthamiana* leaves transiently expressing a combination of LjOSC, CYP716A12_D122Q with or without LjCPR2 using pBYR2HS. A combination of LjOSC, LjCPR2, and empty vector was used as a background control. β-Amyrin (**1**), erythrodiol (**2**), and oleanolic acid (**3**) were used as authentic standards. Uvaol (*) was used as an internal standard. **(B)** Quantification of triterpene concentration in *N*. *benthamiana* leaf extracts. Data are representative of at least three biological replicates (*n* = 3) and are expressed as the mean ± SEM. Differences in oleanolic acid production were examined using Student’s *t*-test for each sample. ^*^p < 0.05; ND, not detected.

CYPs require electrons transferred by NADPH-CPR for site-specific oxidation. In a heterologous host, native CPRs are likely to be insufficient partners for heterologous CYPs to achieve optimal performance (Zhu et al., 2018; Liu et al., 2019, 2021; Theron et al., 2019; Istiandari et al., 2021). To evaluate the efficacy of co-expression of additional CPR in our system, equal volumes of *A*. *tumefaciens* expressing LjOSC1, CYP716A12_D122Q with or without additional LjCPR2 were mixed and infiltrated into *N*. *benthamiana*. A mixture of *A*. *tumefaciens* harboring pBYR2HS expressing LjOSC1, LjCPR2, and an empty vector was used as a background control. GC–MS analysis and quantification of oleanolic acid in leaf extracts were performed (Figure 2). The *N*. *benthamiana* leaves transiently expressing LjOSC1 and CYP716A12_D122Q with LjCPR2 produced 39.3 ± 3.4 mg dw oleanolic acid, which was 2.7-fold higher than that without LjCPR2 co-expression (14.7 ± 0.3 mg/g dw; Figure 2B).

These results indicate that additional CPR co-expression is effective to improve the microenvironment and achieve optimal performance of heterologous CYPs in *N*. *benthamiana*, leading to increasing oleanolic acid production.

### Application of ascorbic acids for higher rates of oleanolic acid production

**Figure 3:**
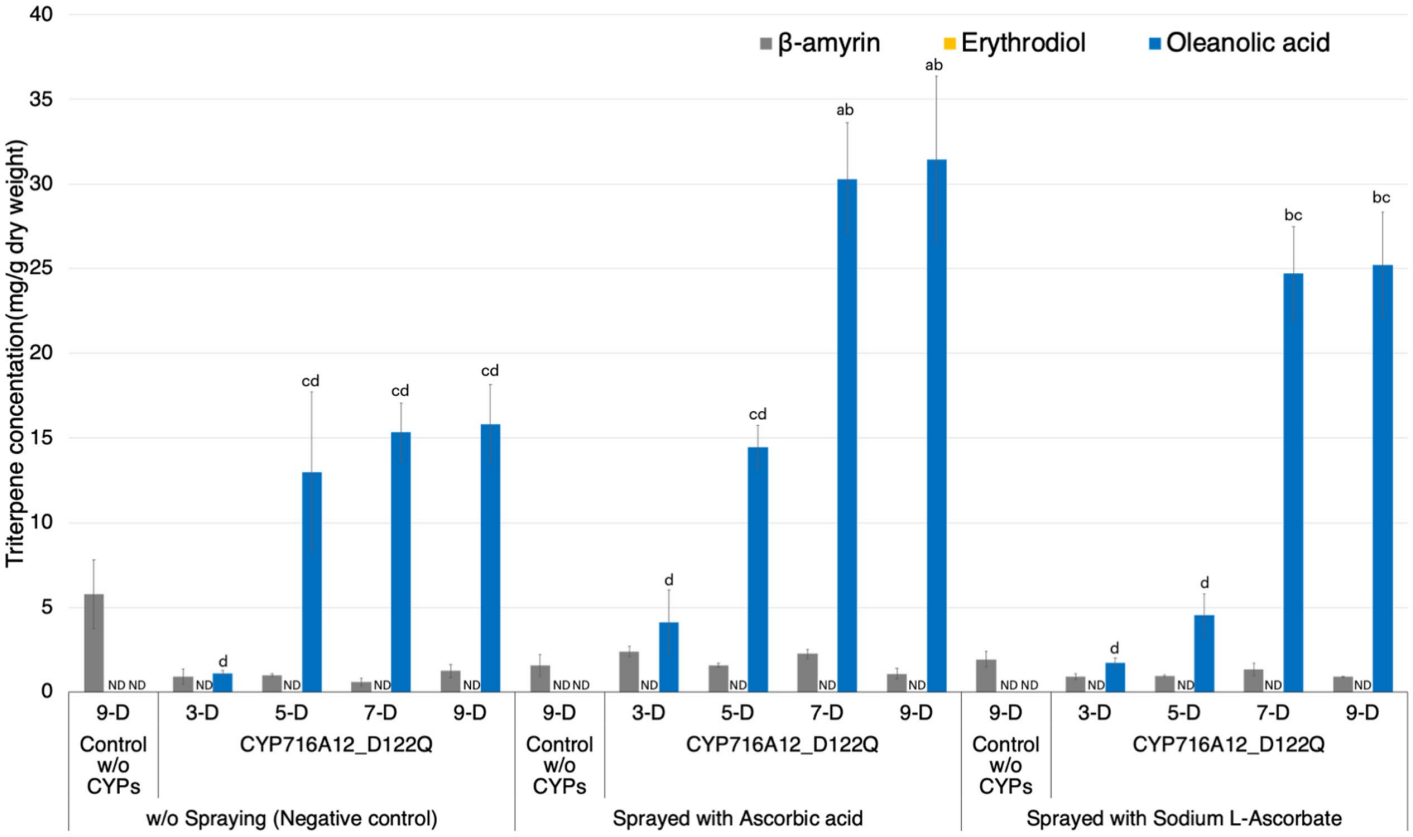
Improving production of oleanolic acid in *Nicotiana benthamiana* leaves by application of ascorbic acid. Quantification of triterpene concentration in *N*. *benthamiana* leaf extracts transiently expressing a combination of LjOSC, LjCPR2, and CYP716A12_D122Q or pBYR2HS empty vector (control) with or without foliar application of 200 mM ascorbic acid or ascorbic acid sodium salt (sodium l-ascorbate). Metabolites were extracted from leaves at 3, 5, 7, or 9 days after agroinfiltration. A combination of pBYR2HS expressing LjOSC, LjCPR2, and empty vector was used as a background control (9 days). Values and error bars represent the mean and standard deviation and the data are representative of at least three biological replicates (*n* = 3). Letters indicate significant differences in the levels of oleanolic acid (a–d) among samples (one-way ANOVA with Tukey’s *post-hoc* test, *p* < 0.05). ND, not detected.

Agrobacterium-induced leaf necrosis, referring to the death of leaves, can occur in *N*. *benthamiana* leaves after infiltration (Nosaki et al., 2021). To prevent necrosis after agroinfiltration, leaves were sprayed with ascorbic acid, and optimal incubation times for improving oleanolic acid production were determined. The leaves were infiltrated with a mixture of equal volumes of *A*. *tumefaciens* harboring pBYR2HS expressing LjOSC1, LjCPR2, and CYP716A12_D122Q. *A*. *tumefaciens* harboring pBYR2HS expressing LjOSC1, LjCPR2, and empty vector was used as the background control. Leaves were sprayed with 200 mM ascorbic acid or 200 mM ascorbic acid sodium salt (sodium l-ascorbate) every 2 days (i.e., days 1, 3, 5, and 7) post-infiltration. Leaves that were not sprayed with ascorbic acid were used as negative controls. The leaves of three plants were harvested for triterpenoid analyses at 3, 5, 7, and 9 days post-infiltration. Extracts of leaves were subjected to GC–MS analysis and triterpenoid quantification (Figure 3; Supplementary Figure 4). Oleanolic acid production was significantly increased by spraying leaves with 200 mM ascorbic acid (30.3 ± 3.3 mg/g dw) or 200 mM sodium l-ascorbate (24.7 ± 2.8 mg/g dw) after 7 days of agroinfiltration compared with without spraying ascorbic acid (15.3 ± 1.7 mg/g dw). No significant difference was observed in the yield detected at 7 and 9 days after infiltration (Figure 3).

As a result, foliar spraying with 200 mM ascorbic acid and incubation for 7 days after agroinfiltration yielded the highest oleanolic acid production titer, so these conditions were used in further experiments.

### Identification of CYP716A12 mutants with the highest potential for oleanolic acid production

**Figure 4:**
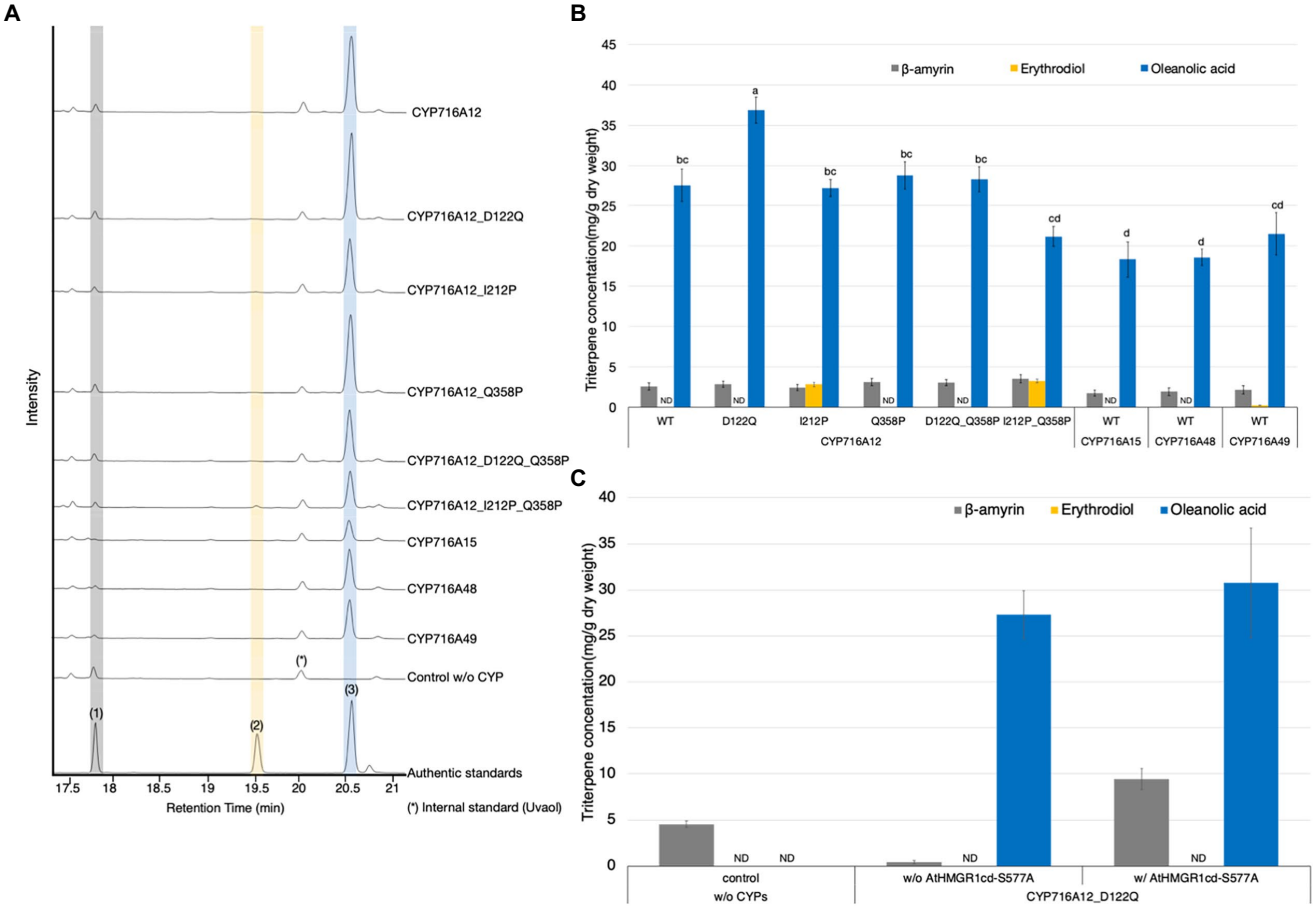
Identification of CYP716A with the highest potential for high-level oleanolic acid production in *Nicotiana benthamiana* using the Tsukuba system. **(A)** TICs of extracts from *N*. *benthamiana* leaves transiently expressing a combination of LjOSC, LjCPR2, and mutant CYP716A12s (D122Q, I212P, Q358P, D122Q Q358P, and I212P Q358P), wild-type CYP716As (CYP716A12, CYP716A15, CYP716A48, and CYP716A49), or empty vector as a control. β-Amyrin (**1**), erythrodiol (**2**), and oleanolic acid (**3**) were used as authentic standards. Uvaol (*) was used as an internal standard. **(B)** Quantification of triterpene concentration in *N*. *benthamiana* leaf extracts analyzed in **(A)**. **(C)** The accumulation of triterpenes in *N*. *benthamiana* leaves transiently expressing a combination of LjOSC1, LjCPR2, and CYP716A12_D122Q with or without AtHMGR1cd-S577A. Values and error bars represent the mean and standard deviation, and the data are representative of at least three biological replicates with similar results from three independent experiments (*n* = 9). Letters indicate significant differences in the levels of oleanolic acid (a–d) among samples (one-way ANOVA with Tukey’s *post-hoc* test, *p* < 0.05). ND, not detected.

In a previous study, we identified critical amino acid residues for improving the catalytic activity and substrate specificity of CYP716A for triterpenoid production in *S. cerevisiae* (Romsuk et al., 2022). We chose five mutant CYP716A12s with altered product profiles when critical amino acid residues in the substrate recognition site were replaced, i.e., D122Q, I212P, Q358P, D122Q_Q358P, and I212P_Q358P. We also compared the catalytic activities of wild-type CYP716A12, CYP716A15, CYP716A48, and CYP716A49 in oleanolic acid biosynthesis to selected mutant CYP716A12s. In this experiment, all candidate genes were cloned into the pBYR2HS vector to identify the gene with the highest potential for oleanolic acid production in *N*. *benthamiana* using the Tsukuba system. Equal volumes of *A*. *tumefaciens* harboring a pBYR2HS expressing LjOSC1, LjCPR2, and candidate CYP716A were mixed and infiltrated into *N*. *benthamiana*. As a background control, leaves were infiltrated with a mixture of *A*. *tumefaciens* harboring pBYR2HS expressing LjOSC1, LjCPR2, and empty vector. To determine the triterpenoid profile of *N*. *benthamiana* leaf extract, metabolites were analyzed by GC–MS. The metabolite profiles of *N*. *benthamiana* leaves transiently expressing almost all candidates, but not the background control, showed a single major peak of oxidized triterpenoid. The major peak was identified as oleanolic acid (**3**) based on a comparison with the authentic standard (Figure 4A). Only when CYP716A12_I212P or CYP716A12_I212P_Q358P was transiently expressed, erythrodiol (**2**) was detected as a minor oxidized triterpenoid peak (Figure 4A). The concentrations of triterpenoids were examined in *N*. *benthamiana* leaf extracts transiently expressing each of the five mutant CYP716A12s or wild-type CYP716A12, CYP716A15, CYP716A48, or CYP716A49. The highest oleanolic acid concentration was detected in *N*. *benthamiana* leaves transiently expressing CYP716A12_D122Q (36.9 ± 1.6 mg/g dw; Figure 4B).

In our previous studies, co-expression of the *A*. *thaliana* HMGR1 catalytic domain with the S577A mutation (AtHMGR1cd-S577A, lacking an inactivating phosphorylation site and exhibiting better activity *in vitro* compared with AtHMGR1cd) increased triterpenoid production in agroinfiltrated *N*. *benthamiana* leaves (Robertlee et al., 2018; Srisawat et al., 2019). Therefore, we used this strategy to increase oleanolic acid production using the Tsukuba system. Leaves were infiltrated with a mixture of *A*. *tumefaciens* harboring pBYR2HS expressing LjOSC1, LjCPR2, and CYP716A12_D122Q with or without AtHMGR1cd-S577A. Leaves infiltrated with a combination of *A*. *tumefaciens* lacking CYPs were used as background controls.

The leaves were harvested at 7 days post-agroinfiltration and processed for metabolite extraction, GC–MS analysis, and quantification of oleanolic acid (Figure 4C; Supplementary Figure 5). As a result, the accumulation of β-amyrin was increased nearly 21-fold after co-expression of AtHMGR1cd-S577A (Figure 4C). The accumulation of oleanolic acid also tended to increase after co-expression of AtHMGR1cd-S577A (30.8 ± 6.0 mg/g dw; Figure 4C).

### Combinatorial biosynthesis of maslinic acid in *Nicotiana benthamiana*

**Figure 5:**
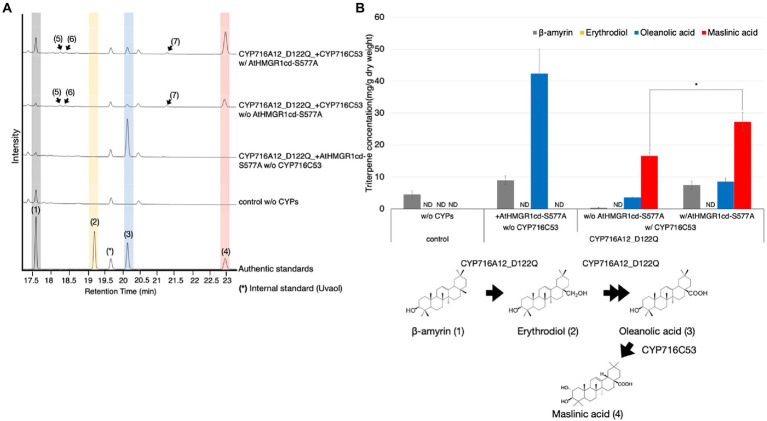
Production of maslinic acid in *Nicotiana benthamiana* leaves using the Tsukuba system. **(A)** TICs of extracts from *N*. *benthamiana* leaves transiently expressing a combination of LjOSC, LjCPR2, CYP716A12_D122Q, and CYP716C53 with or without AtHMGR1cd-S577A. A combination of pBYR2HS expressing LjOSC, LjCPR2, and empty vector was used as a background control. β-Amyrin (**1**), erythrodiol (**2**), oleanolic acid (**3**), and maslinic acid (**4**) were used as authentic standards. Uvaol (*) was used as an internal standard. **(B)** Quantification of maslinic acid concentration in *N*. *benthamiana* leaf extract. Data are representative of at least three biological replicates (*n* = 3) and are expressed as the mean ± SEM. Differences in maslinic acid concentration were compared using Student’s *t*-test for each sample. ^*^p < 0.05; ND, not detected.

We hypothesized that our transient expression platform using the Tsukuba system would be suitable for high-titer production of oleanolic acid as a precursor of other bioactive triterpenoids. There are numerous natural sources of maslinic acid, a pentacyclic triterpene that has been used in traditional medicine (Lozano-Mena et al., 2014; Reyes-Zurita et al., 2016). We previously identified CYP716C53 from gray mangrove (*Avicennia marina*) showed that it can catalyze the C-2α hydroxylation of oleanolic acid to produce maslinic acid in engineered yeast (Nakamura et al., 2018). To produce maslinic acid in *N*. *benthamiana* leaves using the Tsukuba system, leaves were infiltrated with *A*. *tumefaciens* harboring pBYR2HS expressing LjOSC1, LjCPR2, CYP716A12_D122Q, and CYP716C53 with or without AtHMGR1cd-S577A. The leaves were harvested 7 days after agroinfiltration and processed for metabolite extraction. We performed GC–MS analysis and quantification of maslinic acid in leaf extracts (Figure 5). Maslinic acid (**4**) was detected, based on comparison with the authentic standard, as a major peak of oxidized triterpenoid in *N*. *benthamiana* leaves, only when CYP716C53 was transiently expressed. Three new minor peaks (peaks 5, 6, and 7) were also found when CYP716C53 was expressed, although the chemical structures of these peaks are unknown. The mass spectrometry data suggested that peaks 5 and 7 are likely isomers of maslinic acid differing in the position of the hydroxyl group introduced by CYP716C53 from C-2. Peak 6 was presumed to be a mono-hydroxylated product of β-amyrin (Figure 5A; Supplementary Figure 6).

Maslinic acid production was quantified to allow comparison of the condition with versus without AtHMGR1cd-S577A (Figure 5B). *Nicotiana benthamiana* leaves transiently expressing LjOSC1, LjCPR2, CYP716A12_D122Q, and CYP716C53 without AtHMGR1cd-S577A showed maslinic acid production of 16.6 ± 1.0 mg/g dw, which was increased 1.6-fold (27.2 ± 3.0 mg/g dw) after co-expressing AtHMGR1cd-S577A (Figure 5B).

## Discussion

Plants are abundant sources of the bioactive pentacyclic triterpenoids. For example, fruits and leaves of the olive tree (*O. europaea*) are particularly rich sources of oleanolic and maslinic acids. Both oleanolic and maslinic acids are concentrated on the surface of olive leaves to form a physical barrier that is involved in plant defense against water loss and pathogens (Chen et al., 2005; Jager et al., 2009; Romero et al., 2010; Gudoityte et al., 2021). Several recent studies have demonstrated that oleanolic acid has a diverse range of biological activities, including antitumor, antidiabetic, antioxidant, cardioprotective, neuroprotective, antiparasitic, and growth-stimulating properties. Moreover, oleanolic acid is a precursor of several commercially important bioactive triterpenoids (Liu, 1995; Jeon et al., 2019; Kim et al., 2021). CYP716A12 catalyzes the three-step oxidation of C-28 of β-amyrin to produce oleanolic acid *via* erythrodiol (28-hydroxy-β-amyrin) and oleanolic aldehyde intermediates (Fukushima et al., 2011; Suzuki et al., 2018). In a previous study using yeast engineered to produce β-amyrin, we found that the product profile of CYP716A12 was changed by substituting key amino acid residues on substrate recognition sites; three mutant CYP716A12s (D122Q, Q358P, D122Q_Q358P) had potential for high-level oleanolic acid production, and two mutant CYP716A12s (I212P, I212P_Q358P) had potential for high-level erythrodiol production in yeast-producing β-amyrin (Romsuk et al., 2022).

Significant progress in triterpenoid production has recently been reported using microorganisms, particularly *S. cerevisiae*, as alternative hosts (for a review, see Yao et al., 2020). However, when using such microbial systems, extensive metabolic engineering to maximize upstream carbon flux flow from acetyl-CoA to 2,3-oxidosqualene and improving enzyme activities by codon optimization are generally required to achieve high-yield production. Compared with microbial expression systems, *Nicotiana* species provide an optimal environment for plant-derived biosynthetic enzymes. A plant chassis is a rich source of precursor molecules, intermediates from primary metabolism, and enzyme cofactors required to facilitate the functioning of biosynthetic pathways. Moreover, expression of multiple biosynthetic enzymes can be achieved simply by coinfiltration of multiple *A. tumefaciens* strains, each containing a unique construct, without the need to generate multi-gene constructs (Christ et al., 2019; De La Pena and Sattely, 2021).

Using *N*. *benthamiana* as a heterologous expression host for rapid, increased production and reconstruction of plant triterpenoids pathways is a translational synthetic biology platform because it is susceptible to the *Agrobacterium*-based transient expression system, which is scalable and flexible (Geisler et al., 2013; Reed et al., 2017; Reed and Osbourn, 2018). The Tsukuba system is one of the most powerful transient protein expression systems for plant cells, utilizing agroinfiltration to deliver desired genes into plant cells for rapid recombinant protein production based on the use of a geminiviral replication machinery (Yamamoto et al., 2018; Suzaki et al., 2019). Heterologous expression in *N*. *benthamiana* utilizing the Tsukuba system showed that mutant CYP716A12 had the potential for high-level oleanolic acid production. The highest oleanolic acid production titer was found with the mutant CYP716A12 where aspartic acid 122 in substrate recognition site (SRS) I was substituted with glutamine (D122Q). Using this expression system, we demonstrate that oleanolic acid production was increased 13.1-fold (27.3 ± 2.6 mg/g dw) compared with the conventional binary vector (2.1 ± 0.7 mg/g dw; Figure 1B).

As mentioned above, CYP716A12_D122Q showed the highest production in *N*. *benthamiana*, although CYP716A12_D122Q_Q358P showed the highest production in yeast-producing β-amyrin, followed by CYP716A12_D122Q. This might be due to differences in the expression systems, which may affect the catalytic activity (Czarnotta et al., 2017), resulting in different oxidized product yields. Erythrodiol was detected as a minor product only when mutant CYP716A12s, in which the key amino acid residue isoleucine 212 (SRSII) was substituted with proline (I212P or I212P_Q358P). Isoleucine 212 and the nearest-neighbor residues in SRSII of CYP716A12 significantly influenced the efficiency of C-28 oxidation of triterpene backbones. By substituting these residues in the ligand-binding pocket, it is possible to alter the production of triterpene-oxidized products in *N*. *benthamiana*.

In this study, we also compared *N*. *benthamiana* and *N. tabacum* as hosts for the production of oleanolic acid. Although there were no significant differences in the production of β-amyrin between *N. tabacum* and *N*. *benthamiana* leaves (Figure 1C), the levels of oleanolic acid production were 3.3-fold higher in the leaves of *N*. *benthamiana* than in those of *N. tabacum*. These observations would thus tend to imply that in terms of product yield per leaf, *N. benthamiana* might be a more appropriate host for producing oleanolic acid. Biological stress caused by *Agrobacterium* infection causes necrosis and/or dehydration, reducing the transformation and expression efficiency of recombinant proteins (Herlache et al., 2001; Gils et al., 2005; Magdum, 2013). Spraying leaves after agroinfiltration with sodium l-ascorbate reduced the necrosis of leaves and increased the accumulation of human recombinant proteins transiently expressed in *N*. *benthamiana* (Nosaki et al., 2021). Our results indicate that spraying with 200 mM ascorbic acid after agroinfiltration led to an almost two-fold increase in oleanolic acid accumulation at 7–9 days after agroinfiltration (Figure 3).

Triterpenoids are structurally diverse because of modification of the triterpene scaffold by CYPs. These enzymes require electrons transferred by NADPH-CPR to catalyze site-specific oxidation (Istiandari et al., 2021). Triterpenoid production can be enhanced when CYPs are in a microenvironment that supports their optimal performance (Zhu et al., 2018; Liu et al., 2019, 2021; Theron et al., 2019; Istiandari et al., 2021). In this study, we observed that oleanolic acid production increased 2.70-fold after co-expression with LjCPR2 (Figure 2B). Our previous research also showed that the co-expression of CPRs derived from certain plant species may or may not be the best partners for CYPs in yeast (Istiandari et al., 2021). Therefore, the proper pairing of CYPs and CPRs would be critical for high-yield triterpenoid production in *N*. *benthamiana*. In addition, to achieve optimal CYP activity, future studies must determine the best CPR:CYP ratio.

In a previous report, the triterpenoid 12,13-epoxy,16-hydroxy-β-amyrin was obtained at a yield of 1.18 mg/g dw from leaves following transient expression in *N*. *benthamiana* (Geisler et al., 2013; Reed et al., 2017). Moreover, co-expression of the HMGR catalytic domain increased triterpenoid production; the levels of simple (β-amyrin) and oxidized triterpenoid (i.e., 12,13-epoxy,16-hydroxy-β-amyrin) production reached 3.3 and 3.9 mg/g dw, respectively (Reed et al., 2017; Reed and Osbourn, 2018). In our previous studies using a conventional binary vector, we also found enhanced α-amyrin production in *N*. *benthamiana* after transient co-expression of AtHMGR1cd with the S577A mutation (AtHMGR1cd-S577A) lacking the inactivating phosphorylation site (Robertlee et al., 2018; Srisawat et al., 2019). In the present study, we demonstrated that transient AtHMGR1cd-S577A expression increased triterpenoid production in *N*. *benthamiana* using the Tsukuba system. After transient co-expression of AtHMGR1cd-S577A, the amounts of both simple and oxidized triterpenoids were increased, yielding β-amyrin (9.4 mg/g dw, Figure 4C), oleanolic acid (30.8 mg/g dw, Figure 4C), and maslinic acid (27.2 mg/g dw, Figure 5B). Therefore, our transient expression system in *N*. *benthamiana* showed improved triterpenoid production compared with the previously reported yield.

Maslinic acid, a pentacyclic triterpene that has been used in traditional medicine for centuries, has antitumor, antidiabetic, antioxidant, cardioprotective, antiparasitic, and growth-stimulating properties (Jager et al., 2009; Romero et al., 2010; Reyes-Zurita et al., 2016). In our previous *in vivo* functional analysis of CYP716C53 in engineered yeast, maslinic acid was produced as a minor product when co-expressed with bAS, CPR, and CYP716A259 (β-amyrin C-28 oxidase of *A. marina*; Nakamura et al., 2018). In comparison, maslinic acid was detected as a major oxidized product when CYP716C53 was expressed in *N*. *benthamiana* leaves using the Tsukuba system (Figure 5). Maslinic acid is found in various natural sources, including olives, which are a rich source of maslinic acid (Romero et al., 2010; Lozano-Mena et al., 2014). The accumulation of triterpenoids in raw plant materials may also depend on the age of the plant (Jager et al., 2009; Romero et al., 2010; Reyes-Zurita et al., 2016). In this study, maslinic acid was produced in *N*. *benthamiana* leaves, yielding 27.2 ± 3.0 mg/g dw at 7 days after agroinfiltration (Figure 5B). This concentration is 20.7 times higher than that in olive fruit (cv. Kalamata), which have been reported to contain high maslinic acid concentration (1.3 mg/g dw; Romero et al., 2010; Lozano-Mena et al., 2014).

In conclusion, this study established a framework for optimizing oleanolic acid production as a precursor of bioactive triterpenoids *via* Agrobacterium-mediated transient expression in *N*. *benthamiana* using the Tsukuba system. We confirmed the utility of this approach by producing substantial amounts of maslinic acid, as an example of a pharmaceutically active plant-derived triterpenoid. This research will contribute to the development of a more efficient platform for synthesizing and accessing previously unavailable natural products and analogs, as well as the possibility of revitalizing drug discovery pipelines.

## Data availability statement

The original contributions presented in the study are included in the article/Supplementary material, further inquiries can be directed to the corresponding author.

## Author contributions

JR, SY, TM, and HS conceived the study. JR, KM, and HS designed the experiment. SY, EF, TM, and HS supervised the study. JR performed the experiments and wrote the manuscript. JR and HS analyzed the data. KM and HS made manuscript revisions. All authors contributed to the article and approved the submitted version.

## Funding

This work was supported by JSPS KAKENHI Grant Numbers JP19H04657, JP20H02913, and JP21K19082 awarded to HS, Osaka University’s International Joint Research Promotion Program (Type B) awarded to EF and TM, and by the Monbukagakusho Scholarship from MEXT awarded to JR.

## Conflict of interest

The authors declare that the research was conducted in the absence of any commercial or financial relationships that could be construed as a potential conflict of interest.

## Publisher’s note

All claims expressed in this article are solely those of the authors and do not necessarily represent those of their affiliated organizations, or those of the publisher, the editors and the reviewers. Any product that may be evaluated in this article, or claim that may be made by its manufacturer, is not guaranteed or endorsed by the publisher.
