# Supplementary material for: High-yield bioactive triterpenoid production by heterologous expression in Nicotiana benthamiana using the Tsukuba system
Source: Front Plant Sci. 2022 Aug 18;13:991909. doi: 10.3389/fpls.2022.991909 (PMC9447470; doi:10.3389/fpls.2022.991909)
Supplement: Supplementary file 1 [file Data_Sheet_1.pdf]

Supplementary Material

1 Supplementary Data

List of Figures

Supplementary Figure 1. Schematic diagram of the T-DNA region of the conventional binary vector (pYS_015) or gateway-compatible version of pBYR2HS.

Supplementary Figure 2. Standard curves of authentic (A) β-amyrin (**1**), (B) erythrodiol (**2**), (C) oleanolic acid (**3**) and (D) Maslinic acid (**4**).

Supplementary Figure 3. Total ion chromatograms of the extracts from different Nicotiana species

Supplementary Figure 4. TIC of the extracts from *N. benthamiana* leaves with or without application of ascorbic acid after agroinfiltration.

Supplementary Figure 5. TICs of the extracts from *N. benthamiana* leaves transiently expressing a combination of LjOSC1, LjCPR2, CYP716A12_D122Q, and with or without *AtHMGR1*cd-S577A.

Supplementary Figure 6. Mass fragmentation pattern of three minor peaks from the GC profile shown in Figure 5A.

Supplementary Method

1. The protein sequence of the enzymes used in this study

>A_MtCYP716A12_*Medicago_truncatula* (Genbank accession no. ABC59076.1)
MEPNFYLSLLLLFVSFISLSLFFIFYKQKSPLNLPPGKMGYPIIGESLEFLSTGWKGHPEKFIFDRM
RKYSSELFKTSIVGESTVVCCGAASNKFLFSNENKLVTAWWPDSVNKIFPTTSLDSNLKEESIKMRK
LLPQFFKPEALQRYVGVMDVIAQRHFVTHWDNKNEITVYPLAKRYTFLLACRLFMSVEDENHVAKFS
DPFQLIAAGIISLPIDLPGTPFNKAIKASNFIRKELIKIIKQRRIDLAEGTASPTQDILSHMLLTSD
ENGKSMNELNIADKILGLLIGGHDTASVACTFLVKYLGELPHIYDKVYQEQMEIAKSKPAGELLNWD
DLKKMKYSWNVACEVMRLSPPLQGGFREAITDFMFNGFSIPKGWKLYWSANSTHKNAECFPMPEKFD
PTRFEGNGPAPYTFVPFGGGPRMCPGKEYARLEILVFMHNLVKRFKWEKVIPDEKIIVDPFPIPAKD
LPIRLYPHKA

>A_VvCYP716A15_*Vitis_vinifera* (Genbank accession no. BAJ84106.1)
MEVFFLSLLLIFVLSVSIGLHLLFYKHRSHFTGPNLPPGKIGWPMVGESLEFLSTGWKGHPEKFIFD
RISKYSSEVFKTSLLGEPAAVFAGAAGNKFLFSNENKLVHAWWPSSVDKVFPSSTQTSSKEEAKKMR
KLLPQFFKPEALQRYIGIMDHIAQRHFADSWDNRDEVIVFPLAKRFTFWLACRLFMSIEDPAHVAKF
EKPFHVLASGLITVPIDLPGTPFHRAIKASNFIRKELRAIIKQRKIDLAEGKASQNQDILSHMLLAT
DEDGCHMNEMEIADKILGLLIGGHDTASAAITFLIKYMAELPHIYEKVYEEQMEIANSKAPGELLNW
DDVQNMRYSWNVACEVMRLAPPLQGAFREAITDFVFNGFSIPKGWKLYWSANSTHKSPECFPQPENF
DPTRFEGNGPAPYTFVPFGGGPRMCPGKEYARLEILVFMHNVVKRFKWDKLLPDEKIIVDPMPMPAK
GLPVRLHPHKP

>A_OeCYP716A48_*Olea_europaea* (Genbank accession no. BAP59949.1)
MEFFYVSLLCLFVFLISLSLHFLFYKNKSSFSGQIPPGKTGWPVIGESLEFLSNGWKGHPEKFIFDR
IAKYSSYVFRTHLFGEPAAVFCGANGNKFLFSNENKLVQAWWPASVDKVFPSSNQTSSKEEAVKMRK
MLPTFFKPEALQRYVGIMDHIAQRHFSDGWDNKNEVVVFPLAKRYTFWLACRLFVSVEDPAHVAKFA
DPFNELASGLISIPIDLPGTPFHRAIKSSNFIRKELVSIIKQRKIDLAEGKASPTQDILSHMLLTSD
ESGKFMHELDIADKILGLLVGGHDTASSACTFVVKYLAELPEIYEGVYQEQMEIAKSKAPGELLNWD
DIQKMKYSWNVACEVLRLAPPLQGAFREAITDFMFNGFSIPKGWKLYWSANSTHRNSEFFPEPLKFD
PSRFEGSGPAPYTFVPFGGGPRMCPGKEYARLEILVFMHHLVKRFKWEKLIPDEKIVVDPMPIPAKG
LPIRLYPLNA

>A_BvCYP716A49_*Beta_vulgaris* (Genbank accession no. BAP59950.1)
MELFFLCGLILFLSLSLASLYLLYNHNSTKGYRVPPGTMGWPVVGESLEFLSTGWKGYPEKFIFDRL
SKYAPNQIFKTSILGEKVAVICGAAGNKFLYSNENKLVQAWWPSSVDKIFPSSTQTSSKEESKKMRK
LLPNFLKPEALQRYIPIMDTIAIRHMESGWDGKDKVEVFPLAKRYTFWLACRLFLSIEDPDHVAKFA
EPFNDIAAGIISLPVNLPGTPFNRGIKSSNVVRKELRAIIKQRKLDLADGKASTTQDILSHMLLTAD
EDGRFMTEMDIADKILGLLIGGHDTASAACTFVVKYLAELPHVYEAVCKEQMEIAKSKAEGELLNWE
DIQKMKYSWNVACEVMRLAPPLQGGFREAISDFMYGGFQVPKGWKLYWSANSTHRNPECFPEPEKFD
PSRFEGKGPAPYTYVPFGGGPRMCPGKEYARLEILVFMHNVVKRFKWEKVLPNEKVIVNPMPIPENG
LPVRLFPHPQIVAA

>*Lotus japonicus*_cytochrome_P450_reductase (Genbank accession no. BAG68945.1)

MEESSSMKISPLDLMSAMIKGTLDPSNVSSTSGAGSVFLENREFVMVLTTSIAVLIGCVVVFIWRRS
TGNKAKSIEPPKRVVEKLSDEAEVDDGTRKVTIFFGTQTGTAEGFAKAIAEEAKVRYEKAKFKIVDM
DDYAQDDDEYEEKLKKETLALFFLATYGDGEPTDNAARFYKWFLEGDEKEEGWLRNLEYAVFGLGNR
QYEHFNKVAIEVDDKLADFGGKRLVKVGLGDDDQCIEDDFTAWKEELWPALDELLRGDDDTTVSTPY
TAAVLEYRVVIHDPLDASVDEKKWHNVNGHAIVDAQHPVRSNVAVRKELHTPVSDRSCTHLEFDISG
TGVAYETGDHVGVYCENLSETVEEAVRLLGLSPDTYFSVHTDDEDGKPLSGSSLPPTFPPCTLRTAI
ARYADVLSSPKKSVLLALAAHASNPSEADRLRHLASPAGKDEYSEWVIASQRSLLEVMAEFPSAKPP
IGVFFAAIAPRLQPRFYSISSSPRMAPSRIHVTCALVNDKMPTGRIHRGVCSTWMKNSVPLEKSQDC
SWAPIFVRQSNFKLPADNKVPIIMIGPGTGLAPFRGFLQERLALKEDGAELGPSVLFFGCRNRQMDY
IYEDELNHFVNSGALSELIVAFSREGPTKEYVQHKMMEKASDIWNMISQGAYIYVCGDAKGMARDVH
RTLHTILQEQGSLDSSKAEGMVKNLQLNGRYLRDVW

2. The nucleotide sequence of the codon-optimized CDS of LjOSC1 used in this study

>Synthetic construct *Lotus japonicus* OSC1 gene, complete CDS
(Genbank accession no. LC718125)
ATGTGGAAACTTAAAGTTGCTGATGGCGGCAAGGACCCGTACATCTTCTCTACTAACAACTTCGTTG
GCCGGCAGACCTGGGAGTATGATCCTGATGCTGGTACTCCTGAAGAGAGAGCACAGGTTGAAGAAGC
TAGGCAGGACTTCTACAACAACCGGTACAAGGTTAAGCCTTGCGGCGATCTTCTTTGGAGGTTCCAG
GTTCTGCGTGAGAACAACTTCAAGCAGACCATTCCGAGCGTGAAGATCGAGGATGGTGAAGAGATTA
CCTACGAGAAGGCTACCACCACCTTGAAGAGAGCTGCTCATCATCTTGCTGCTCTGCAGACTTCTGA
TGGTCATTGGCCTGCTCAAATTGCTGGCCCTTTGTTTTTCCAGCCTCCTCTGGTTTTCTGCATGTAC
ATTACCGGCCACCTGAACTCTGTGTTCCCTGAAGAGTACCGGAAAGAGATCCTGCGGTACATCTACG
TGCACCAGAATGAAGATGGTGGTTGGGGTCTGCATATTGAGGGACACTCTACTATGTTCTGCACCGC
TCTGAACTACATCTGCATGAGGATGCTTGGTGAGGGTCCTGATGGTGGTCAGGATAATGCTTGTGCT
AGAGCCCGGAAGTGGATTCTTGATCATGGTGGTGTGACCCACATTCCGTCTTGGGGTAAGACCTGGC
TTAGCATTCTTGGCATCTTCGACTGGAAGGGCTCTAACCCTATGCCTCCTGAGTTCTGGATTCTGCC
TTCTTTCCTTCCTATGCACCCTGCCAAGATGTGGTGCTATTGCAGGCTTGTGTACATGCCGATGAGC
TACCTGTACGGCAAGAGATTCGTGGGTCCTATCACTCCTCTTATCCTGCAGCTTCGGGAAGAGTTGT
TCACTCAGCCTTATGAGAAGGTGAACTGGAAGAAAGCAAGGCACCAGTGCGCTAAAGAGGACATCTA
TTACCCTCATCCGCTGATCCAGGATCTGATGTGGGATTCTCTGTACCTGTTCACCGAGCCTTTGCTT
ACTAGGTGGCCTTTCAACAAGCTGGTGAGAGAGAAGGCTCTCGAGGTTACCATGAAGCACATTCACT
ACGAGGACGAGAACAGCCGGTACATCACTATTGGCTGCGTTGAGAAGGTTCTGTGCATGCTTGCTTG
CTGGGTCGAAGATCCTAACGGGGACGCTTTTAAGAAGCACCTTGCTAGGATCCCTGACTACCTTTGG
GTTAGCGAAGATGGCATGTGCATGCAGAGCTTCGGTTCTCAAGAATGGGATGCTGGTTTCGCTGTGC
AGGCTCTTTTGGCTACTAACCTTGTGGATGAGCTGGGTCCTACTCTTGCTAAGGGTCACGACTTCAT
CAAGAAGTCCCAGGTTAGGGATAACCCGAGCGGCGATTTCAAGAATATGCACCGGCACATCTCCAAA
GGCAGCTGGACTTTTTCTGATCAGGACCACGGTTGGCAGGTTTCAGATTGCACTGCTGAGGGTCTTA
AGTGCTGCCTTCTGCTTTCTATGCTCCCTCCTGATATCGTGGGCGAGAAGATGGAACCTGAGTGCCT
TTTCGATAGCGTGAACCTTCTGCTGAGCCTGCAGTCTAAGAAAGGTGGACTTGCTGCTTGGGAACCT
GCTGGTGCTCAAGAGTGGCTTGAGCTTCTTAATCCGACCGAGTTCTTCGCTGACATCGTGGTTGAGC
ATGAGTACGTTGAGTGCACCGGTTCTGCTATTGGTGCTCTTGTGCTGTTCAAGAAGCTGTACCCTGG
CCACCGGAAGAAAGAGATTGAGAACTTCATCAGCGAGGCCGTGAGATTCCTTGAGGATACTCAAACC
GCTGACGGTAGCTGGTATGGTAATTGGGGAGTGTGCTTCACCTACGGCTCTTGGTTTGCTCTTGGTG
GTTTGGCTGCTGCTGGTAAGACTTACGCTAACTGCGCTGCTATCCGGAAGGCTGTGAAGTTCCTTTT
GACTACCCAAAGAGGTGACGGCGGTTGGGGAGAGTCTTATCTGTCAAGCCCGAAGAAGATCTACGTT
CCGTTCGAGGGCAACCGTTCTAATGTGGTTCATACCGCTTGGGCTCTGATGGGTCTTATTCATTCAG
GTCAGGCTGAGAGGGATCCAACACCTCTTCATAGAGCTGCTAAGCTGCTGATCAACAGCCAGCTTGA

AGAAGGCGATTGGCCTCAGCAAGAGATCACTGGTGTGTTCATGAAGAACTGCATGCTGCACTACCCG
ATGTACAGGGACATCTACCCTATGTGGGCTCTCGCTGAGTATAGAAGAAGGGTGCCATTGCCTTCTA
CCGCCGTTTAG

3. Standard curve for quantification of triterpenoids in *Nicotiana* leaf extracts

Standard curves were used to determine the relationship between the quantities of the two compounds. They are used to calculate the value of an unknown amount (triterpene concentration) of metabolite extracted from *N. benthamiana* and *N. tabacum* leaf extracts with respect to the one more easily measured (triterpene standard), which has a known concentration. To quantify the concentrations of triterpenes extracted from leaf extracts, an authentic standard curve of triterpene compounds was constructed, and the relative concentrations were calculated by comparing the peak area of the authentic standard with the peak area of an internal standard. An authentic standard curve was used to plot the relative peak area of the internal standard against a varying but known triterpene concentration. The actual triterpene concentration in leaf extracts was calculated using the standard curve equation.

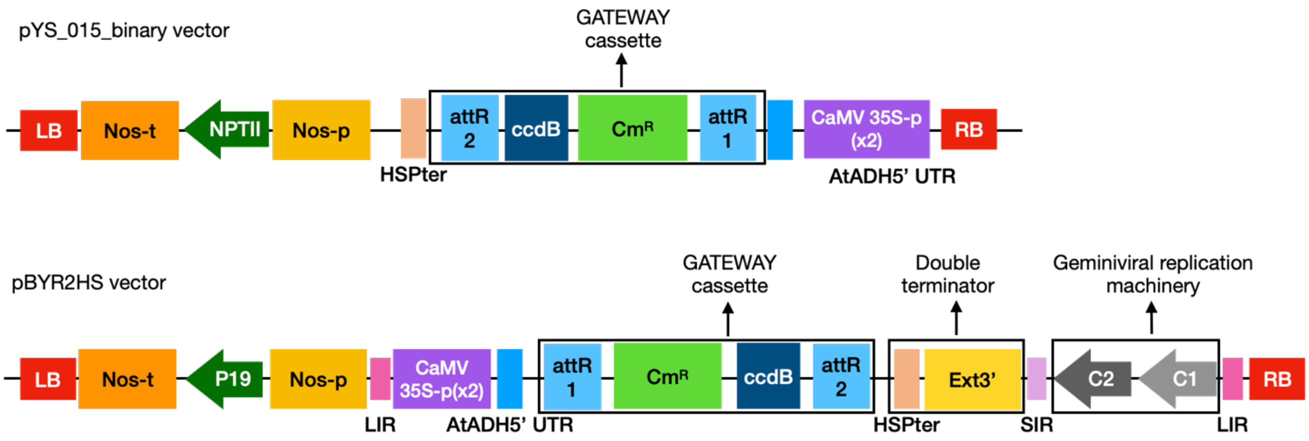

Supplementary Figure 1. Schematic diagram of the T-DNA region of the conventional binary vector (pYS_015) or gateway-compatible version of pBYR2HS.

CaMV 35S-p: Cauliflower mosaic virus (CaMV) 35S promoter with double-enhanced element, AtADH5': 5'-untranslated region (UTR) of *Arabidopsis thaliana* alcohol dehydrogenase gene, HSPter: terminator of heat shock protein gene, Ext3′: tobacco extension gene 3′ element, Nos-t: NOS terminator, LIR: long intergenic region of bean yellow dwarf virus (BeYDV) genome, SIR: short intergenic region of BeYDV genome, C1/C2: BeYDV ORFs C1 and C2 encoding for replication initiation protein (Rep) and RepA, LB and RB: the left and right borders of the T-DNA region, respectively; Nos-p and Nos-t: NOS promoter and terminator, respectively; p19: a gene-silencing suppressor gene from tomato bushy stunt virus; and NPTII: neomycin phosphotransferase II enzyme of *Escherichia coli* Tn5 transposon; attR1 and attR2: attachment sites that allow recombination cloning of the gene of interest from an entry clone; Cm^R: chloramphenicol resistance gene; ccdB gene: a lethal gene that targets DNA gyrase that allows negative selection of plasmid.

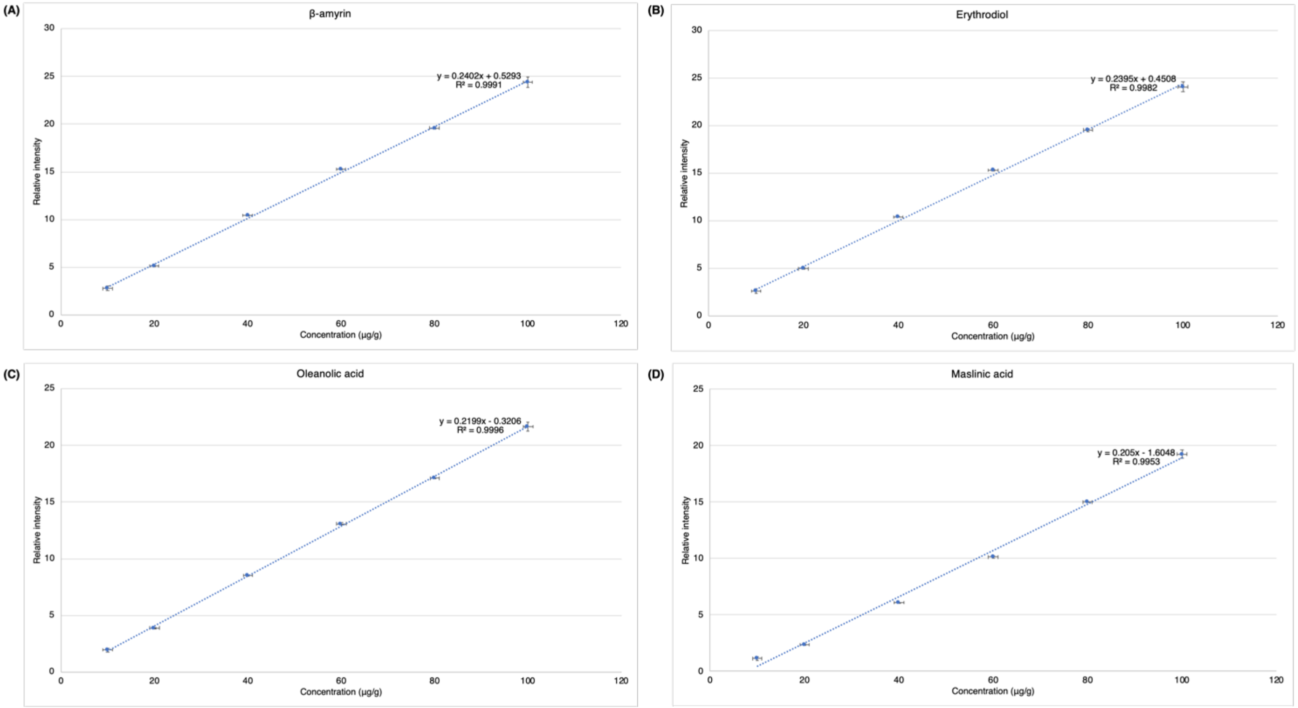

Supplementary Figure 2. Standard curves of authentic (A) β-amyrin (1), (B) erythrodiol (2), (C) oleanolic acid (3) and (D) Maslinic acid (4).

Mean values and standard deviation (error bars) for three replicates are shown.

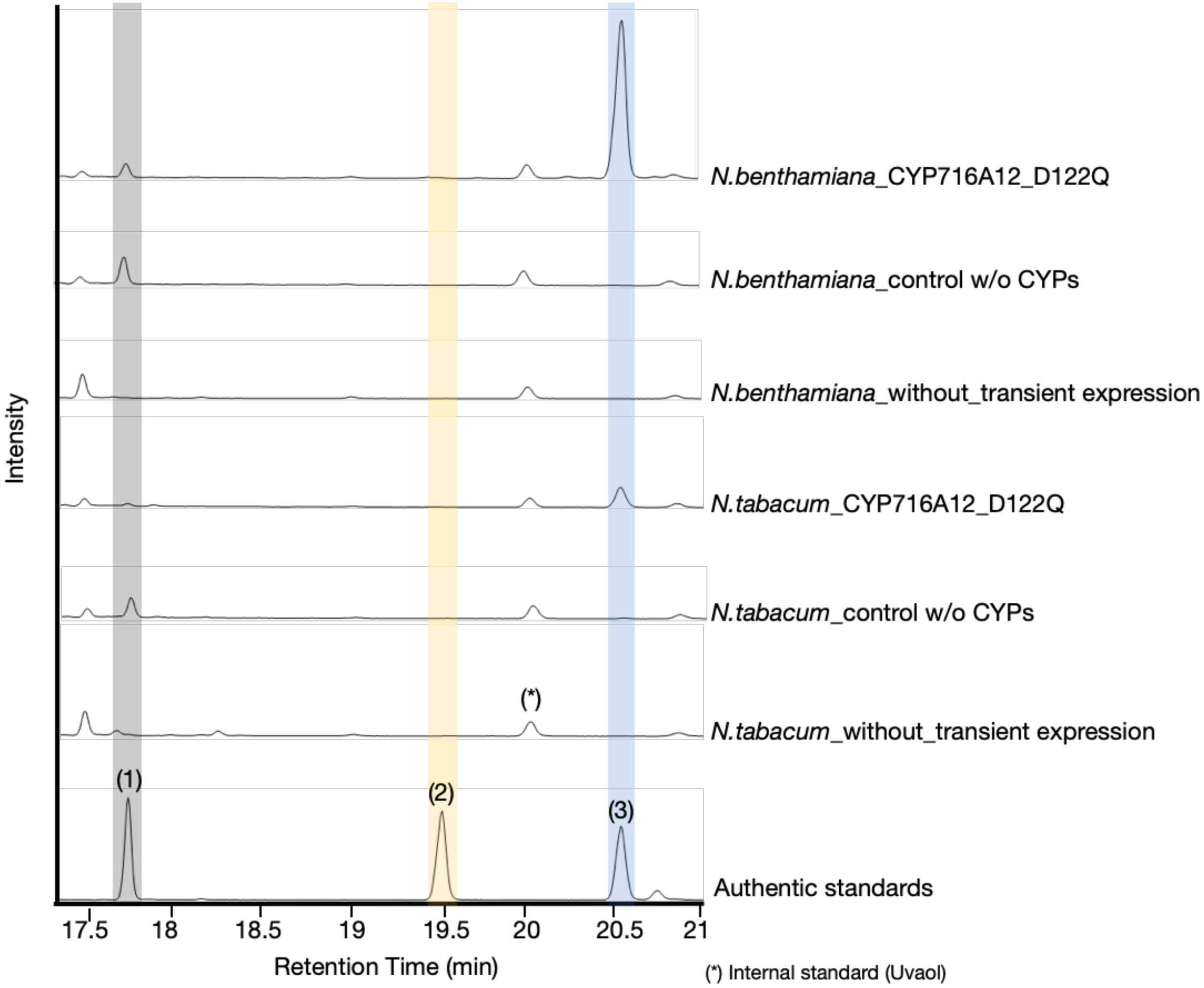

Supplementary Figure 3. Total ion chromatograms of the extracts from different Nicotiana species.

Total ion chromatograms (TICs) of extracts from *N. benthamiana* and *N. tabacum* leaves transiently expressing a combination of LjOSC1, LjCPR2, and with or without CYP716A12_D122Q. As a background control, leaves without transient expression was used. The quantification results are shown in **Figure 1C**. β-Amyrin (**1**), erythrodiol (**2**), and oleanolic acid (**3**) were used as authentic standards. Uvaol (*) was used as an internal standard.

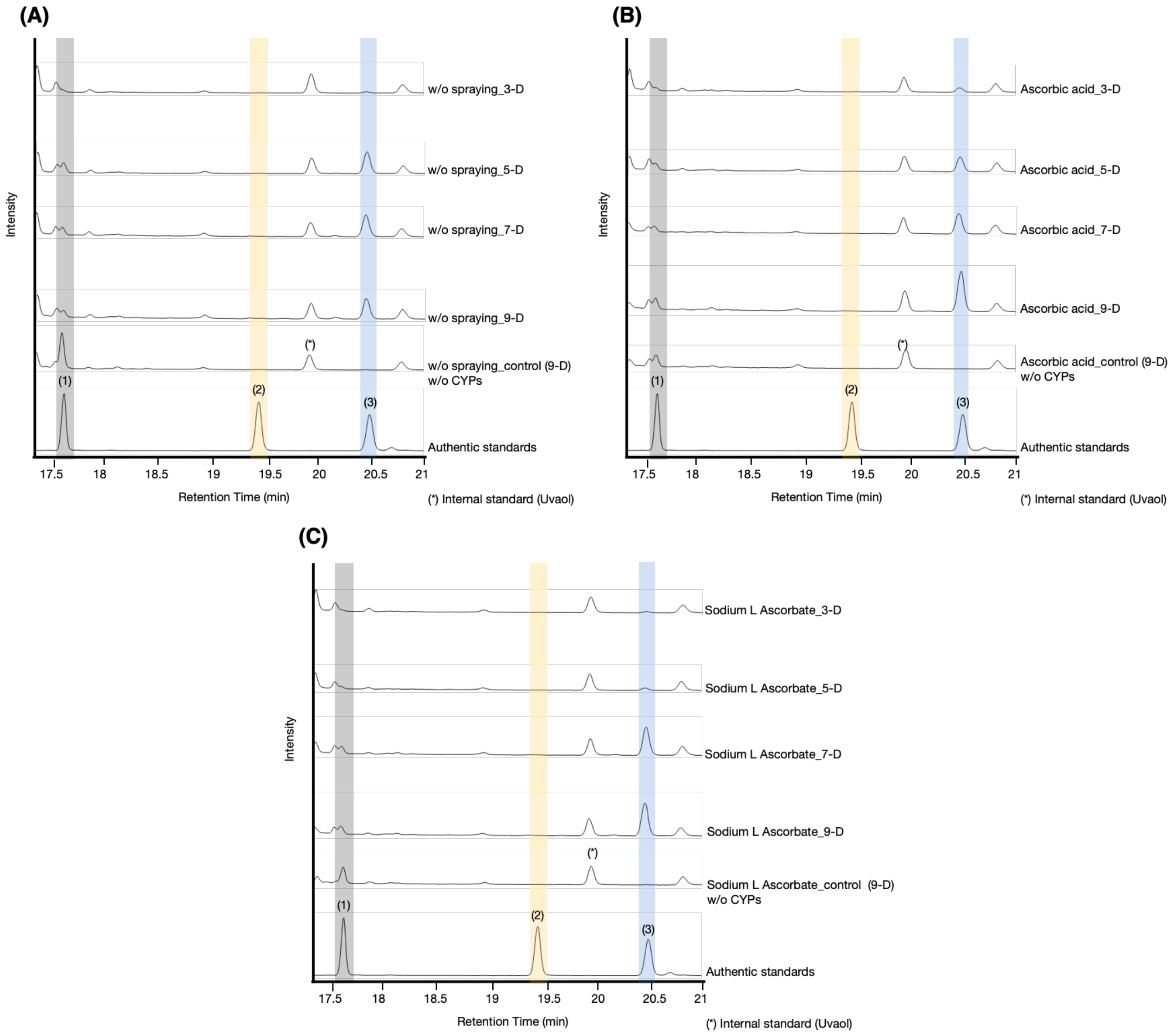

Supplementary Figure 4. TIC of the extracts from *N. benthamiana* leaves with or without application of ascorbic acid after agroinfiltration.

TICs of extracts from *N. benthamiana* leaves transiently expressing a combination of LjOSC, LjCPR2, and CYP716A12_D122Q or pBYR2HS empty vector (w/o CYP control). (A) Leaves without sprayed with ascorbic acid as negative control. (B) Leaves with foliar application of 200 mM ascorbic acid or (C) ascorbic acid sodium salt (sodium L-ascorbate). Metabolites were extracted from leaves at 3, 5, 7, or 9 days after agroinfiltration. The quantification results are shown in **Figure 3**. β-Amyrin (**1**), erythrodiol (**2**), and oleanolic acid (**3**) were used as authentic standards. Uvaol (*) was used as an internal standard.

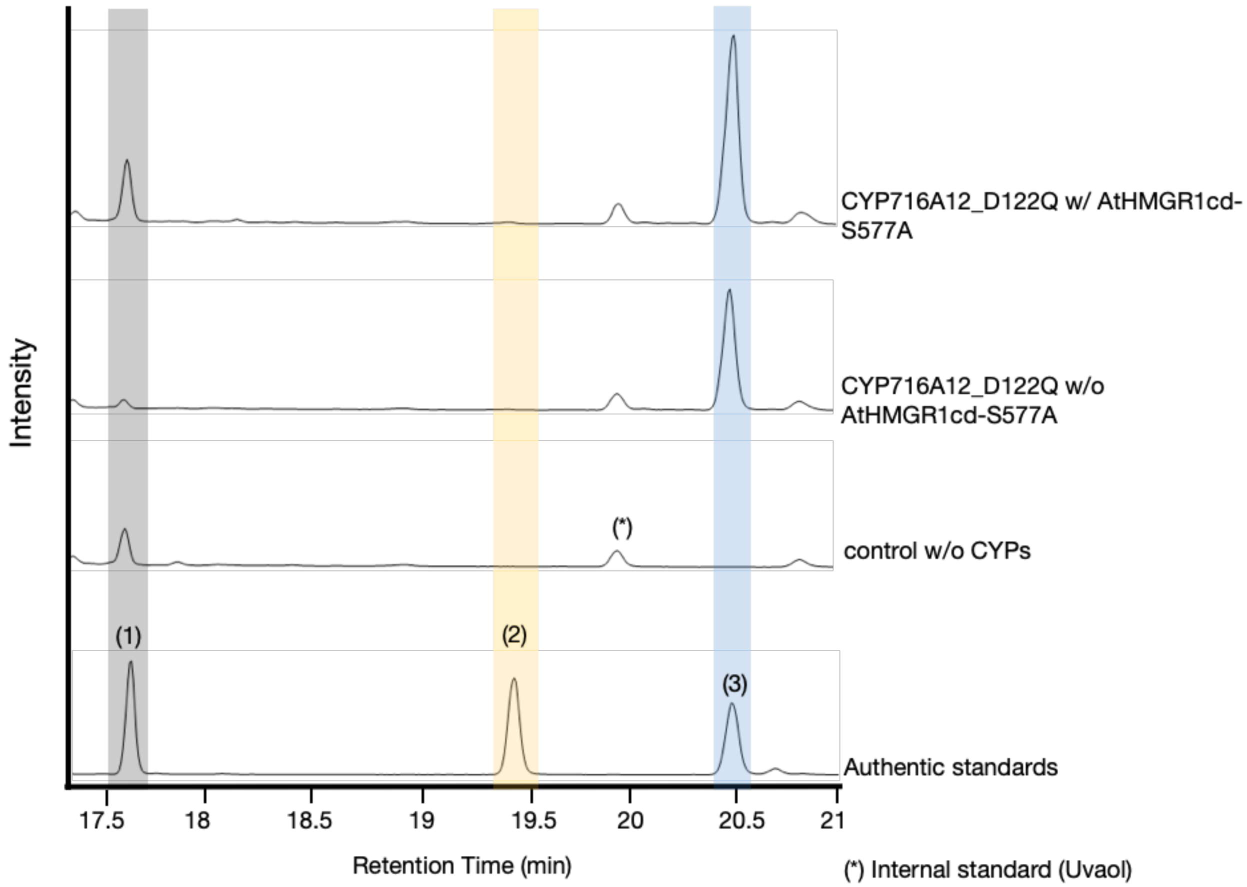

Supplementary Figure 5. TICs of the extracts from *N. benthamiana* leaves transiently expressing a combination of LjOSC1, LjCPR2, CYP716A12_D122Q, and with or without *AtHMGR1*cd-S577A. The quantification results are shown in **Figure 4C**. β-Amyrin (**1**), erythrodiol (**2**), and oleanolic acid (**3**) were used as authentic standards. Uvaol (*) was used as an internal standard.

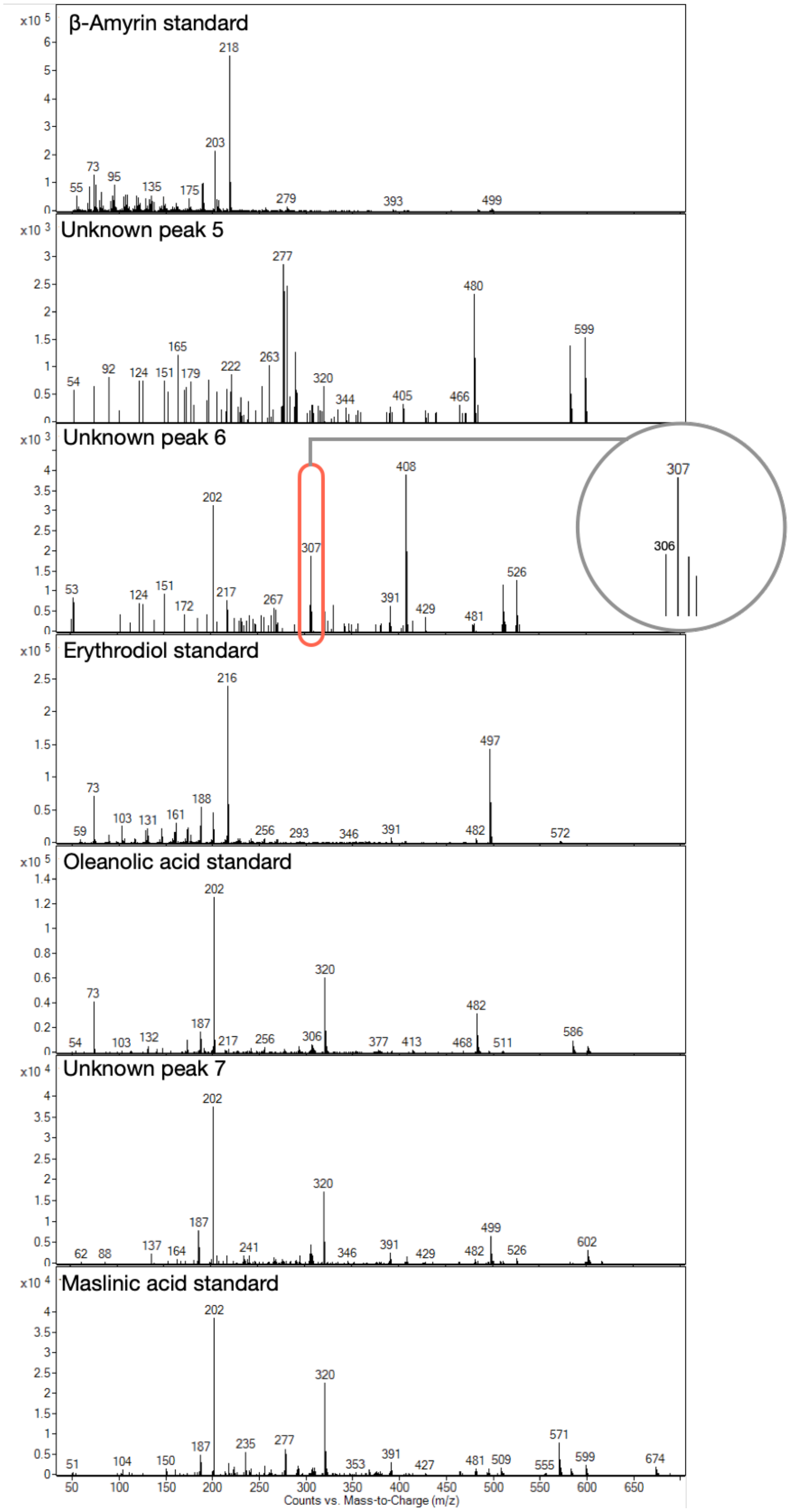

Supplementary Figure 6 Mass fragmentation pattern of three minor peaks from the GC profile shown in Figure 5A.

The mass spectra of peaks 5 and 7 showed fragment ion at m/z 320 (a carboxyl group was added to the C-28 of the β-amyrin backbone), suggesting that both products were derived from oleanolic acid. The mass spectra of peak 6 showed fragment ion at m/z 306, suggesting that one hydroxyl group was introduced into the β-amyrin backbone, probably at C-2.
